# Supplementary material for: Identification of photoperiod-induced specific miRNAs in the adrenal glands of Sunite sheep (Ovis aries)
Source: Front Vet Sci. 2022 Jul 22;9:888207. doi: 10.3389/fvets.2022.888207 (PMC9354845; doi:10.3389/fvets.2022.888207)
Supplement: Supplementary file 1 [file Data_Sheet_1.ZIP › Supplementary file/miRDeep2 file.zip!/miRDeep2 file/Know_Structure/LP42A2/oar-mir-374b.pdf]

miRBase precursor : oar-mir-374b
Total read count : 22721
oar-miR-374b read count : 22091
remaining reads : 630

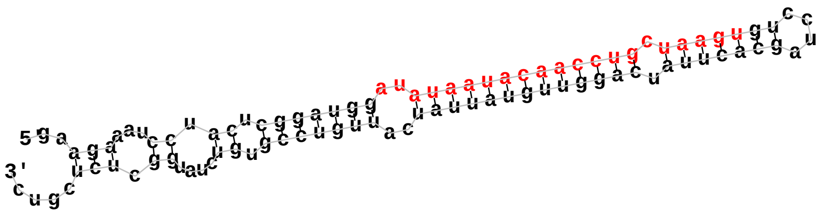

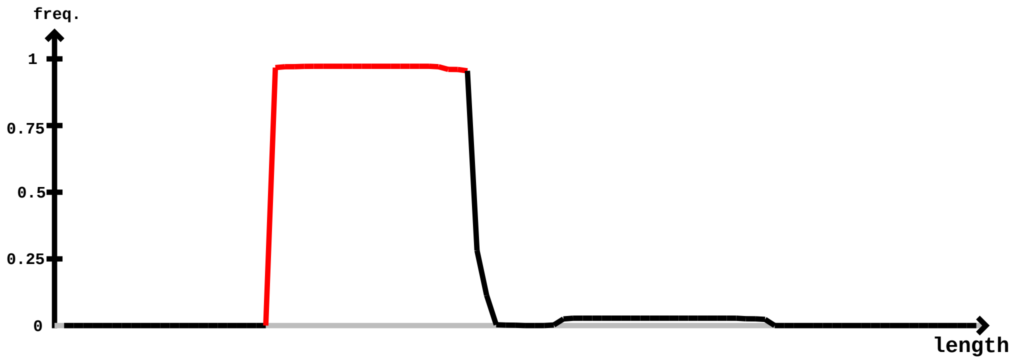


```
                        oar-miR-374b
5'- gaagaaauccuacucggauggauauaauacaaccugcuaaguguccuagcacuuaucagguuguauuaucauuguccgugucuauggcucucguc   -3'    exp
    ((((..((...((.(((((((..(((((((((((((.(((((((....))))))).)))))))))))))..))))))).))..))..)).))...    reads   mm    sample
    .....................auauaauacaaccugAu.........................................................    1       1     seq
    .....................Cuauaauacaaccugcu.........................................................    3       1     seq
    .....................auauaauacaaccugcu.........................................................    38      0     seq
    .....................auauaauacaaccugcua........................................................    223     0     seq
    .....................auauaauacaaccugcuaa.......................................................    9       0     seq
    .....................auauaauacaaccugcuaag......................................................    115     0     seq
    .....................auauaauacaaccugcuaagu.....................................................    15287   0     seq
    .....................auauaauacaaccugcuaagug....................................................    3816    0     seq
    .....................auauaauacaaccugcuaagugu...................................................    2458    0     seq
    .....................auauaauacaaccugcuaaguguc..................................................    24      0     seq
    .....................auauaauacaaccugcuaagugucc.................................................    1       0     seq
    .....................auauaauacaaccugcuaaguguccu................................................    3       0     seq
    ......................uauaauacaaccugcuaagu.....................................................    13      0     seq
    ......................uauaauacaaccugcuaagug....................................................    4       0     seq
    ......................uauaauacaaccugcuaagugu...................................................    49      0     seq
    ......................uauaauacaaccugcuaaguguc..................................................    5       0     seq
    ......................uauaauacaaccugcuaagugucc.................................................    1       0     seq
    ......................uauaauacaaccugcuaaguguccu................................................    1       0     seq
    .......................auaauacaaccugcuaagu.....................................................    3       0     seq
    .......................auaauacaaccugcuaaguguccu................................................    1       0     seq
    ........................uaauacaaccugcuaagu.....................................................    1       0     seq
    ........................uaauacaaccugcuaagugu...................................................    1       0     seq
    ........................uaauacaaccugcuaaguguc..................................................    2       0     seq
    ........................uaauacaaccugcuaagugucc.................................................    7       0     seq
    ........................uaauacaaccugcuaaguguccu................................................    17      0     seq
    .........................aauacaaccugcuaagugucc.................................................    1       0     seq
    .........................aauacaaccugcuaaguguccu................................................    5       0     seq
    ..........................auacaaccugcuaaguguccu................................................    2       0     seq
    .................................................cacuuaucagguuguauuauc.........................    1       0     seq
    .................................................cacuuaucagguuguauuauca........................    1       0     seq
    ..................................................acuuaucagguuguauuauc.........................    31      0     seq
    ..................................................acuuaucagguuguauuauA.........................    1       1     seq
    ..................................................Ccuuaucagguuguauuauc.........................    1       1     seq
    ..................................................acuuaucagguuguauuauca........................    3       0     seq
    ..................................................acuuaucagguuguauuaucau.......................    11      0     seq
```

oar-miR-374b

```
gaagaaauccuacucggauggauauaauacaaccugcuaaguguccuagcacuuaucagguuguauuaucauuguccgugucuauggcucucguc
...................................................cuuaucagguuguauuau..........................   2     0    seq
...................................................cuuaucagguuguauuauc.........................   11    0    seq
...................................................cuuaucagguuguauuauca........................   2     0    seq
...................................................cuuaucagguuguauuauAa........................   1     1    seq
...................................................cuuaucagguuguauuaucau.......................   32    0    seq
...................................................cuuaucagguuguauuaucauu......................   465   0    seq
...................................................cuuaucagguuguauuaucauug.....................   1     0    seq
....................................................uuaucagguuguauuauc.........................   3     0    seq
....................................................uuaucagguuguauuaucau.......................   3     0    seq
....................................................uuaucagguuguauuaucauu......................   59    0    seq
.....................................................uaucagguuguauuaucauu......................   1     0    seq
.....................................................uaucagguuguauuaucauugu....................   1     0    seq
```
